# Virtual Reality Systems for Upper Limb Motor Function Recovery in Patients With Spinal Cord Injury: Systematic Review and Meta-Analysis

**DOI:** 10.2196/22537

**Published:** 2020-12-03

**Authors:** Amaranta De Miguel-Rubio, M Dolores Rubio, Alvaro Alba-Rueda, Alejandro Salazar, Jose A Moral-Munoz, David Lucena-Anton

**Affiliations:** 1 Department of Nursing, Pharmacology and Physiotherapy University of Cordoba Cordoba Spain; 2 Department of Cell Biology, Physiology and Immunology University of Cordoba Cordoba Spain; 3 Department of Statistics and Operational Research University of Cadiz Cadiz Spain; 4 Institute of Research and Innovation in Biomedical Sciences of the Province of Cadiz (INiBICA) University of Cadiz Cadiz Spain; 5 The Observatory of Pain University of Cadiz Cadiz Spain; 6 Department of Nursing and Physiotherapy University of Cadiz Cadiz Spain

**Keywords:** virtual reality, spinal cord injuries, neurological rehabilitation, motor function, physical therapy

## Abstract

**Background:**

Patients with spinal cord injury (SCI) usually present with different motor impairments, including a deterioration of upper limb motor function (ULMF), that limit their performance of activities of daily living and reduce their quality of life. Virtual reality (VR) is being used in neurological rehabilitation for the assessment and treatment of the physical impairments of this condition.

**Objective:**

A systematic review and meta-analysis was conducted to evaluate the effectiveness of VR on ULMF in patients with SCI compared with conventional physical therapy.

**Methods:**

The search was performed from October to December 2019 in Embase, Web of Science, Cumulative Index to Nursing and Allied Health Literature (CINAHL), Scopus, Medline, Physiotherapy Evidence Database (PEDro), PubMed, and Cochrane Central Register of Controlled Trials. The inclusion criteria of selected studies were as follows: (1) comprised adults with SCI, (2) included an intervention with VR, (3) compared VR intervention with conventional physical therapy, (4) reported outcomes related to ULMF, and (5) was a controlled clinical trial. The Cochrane Collaboration’s tool was used to evaluate the risk of bias. The RevMan 5.3 statistical software was used to obtain the meta-analysis according to the standardized mean difference (SMD) and 95% CIs.

**Results:**

Six articles were included in this systematic review. Four of them contributed information to the meta-analysis. A total of 105 subjects were analyzed. All of the studies used semi-immersive or nonimmersive VR systems. The statistical analysis showed nonsignificant results for the Nine-Hole Peg Test (SMD –0.93, 95% CI –1.95 to 0.09), muscle balance test (SMD –0.27, 95% CI –0.82 to 0.27), Motricity Index (SMD 0.16, 95% CI −0.37 to 0.68), Jebsen-Taylor Hand Function Test (JTHFT) subtests (writing, SMD –0.10, 95% CI –4.01 to 3.82; simulated page turning, SMD –0.99, 95% CI –2.01 to 0.02; simulated feeding, SMD –0.64, 95% CI –1.61 to 0.32; stacking checkers, SMD 0.99, 95% CI –0.02 to 2.00; picking up large light objects, SMD –0.42, 95% CI –1.37 to 0.54; and picking up large heavy objects, SMD 0.52, 95% CI –0.44 to 1.49), range of motion of shoulder abduction/adduction (SMD –0.23, 95% CI –1.48 to 1.03), shoulder flexion/extension (SMD 0.56, 95% CI –1.24 to 2.36), elbow flexion (SMD –0.36, 95% CI –1.14 to 0.42), elbow extension (SMD –0.21, 95% CI –0.99 to 0.57), wrist extension (SMD 1.44, 95% CI –2.19 to 5.06), and elbow supination (SMD –0.18, 95% CI –1.80 to 1.44). Favorable results were found for the JTHFT subtest picking up small common objects (SMD –1.33, 95% CI –2.42 to –0.24).

**Conclusions:**

The current evidence for VR interventions to improve ULMF in patients with SCI is limited. Future studies employing immersive systems to identify the key aspects that increase the clinical impact of VR interventions are needed, as well as research to prove the benefits of the use of VR in the rehabilitation of patients with SCI in the clinical setting.

## Introduction

The global estimate of the spinal cord injury (SCI) prevalence is 223 to 755 per million people and the worldwide incidence is 10.4 to 83 per million people per year [1]. SCI produces a high impact on the health care system and society [2]. Patients with SCI usually present with different motor impairments, including a deterioration of upper limb motor function (ULMF), causing an important limitation in the performance of activities of daily living and a loss of quality of life [2,3].

Neurological rehabilitation benefits from virtual reality (VR), which is being used for the assessment and treatment of physical and cognitive impairments, for pain management, and even to acquire surgical skills [4]. This technology is becoming more portable, immersive, and vivid, making it more suitable for a wider range of clinical applications [5]. VR systems allow the creation of virtual environments that can be used to practice, under controlled conditions, different activities that could be hazardous in a real-world setting [6]. Different characteristics such as difficulty, intensity, exposure duration, and feedback can be adjusted to provide personalized experiences [7]. Furthermore, VR and interactive video gaming are presented as a motivational therapy that could increase patient adherence to treatment [8,9]. VR-based interventions are usually provided by commercial devices such as Nintendo Wii [10], PlayStation [11], and Xbox Kinect [12], among others. According to the level of immersion, VR systems can be divided into immersive, semi-immersive, or nonimmersive systems. Immersive systems provide a full integration into the virtual environment that is delivered through head-mounted displays and VR caves. Semi-immersive and nonimmersive systems consist of displaying the environment through a screen and these systems are usually used in video game consoles. Furthermore, VR systems can be combined with different devices such as gloves, electrical stimulation devices, and exoskeletons [13].

Several studies on the use of VR interventions have been carried out in different neurological disorders, such as stroke [14-17], cerebral palsy [18,19], Parkinson disease [20,21], and multiple sclerosis [22-24]. Nevertheless, the odds of a successful recovery are different for each disease, so the results obtained by VR interventions could be different as well. Specifically, patients with SCI usually suffer from significant participation restrictions [25], so the physical treatment should be focused on keeping the residual functionality after SCI [26].

SCI occurs with greater frequency at the cervical and thoracic levels than at lumbosacral levels. Patients with cervical and thoracic SCI can suffer loss of arm and hand function and consequently reduce significantly their autonomy and independence. However, small improvements in arm and hand function could improve the performance of the activities of daily living, independence, and quality of life, and thus recovering ULMF in patients with cervical and thoracic levels of SCI is a primary challenge [3]. In this sense, the scientific evidence through systematic reviews and meta-analyses on the potential use of VR systems to recover ULMF in patients with SCI is limited. de Araújo et al [27] stated that VR therapy could be used to improve motor function. A structured review performed by Yeo et al [28] concluded that VR therapy provides benefits on balance and posture. Conversely, a recent meta-analysis published by our group [29] suggested that VR interventions mat not be effective to improve the functional performance after SCI. Nevertheless, the previous reviews were not restricted specifically to the assessment of ULMF. We hypothesize that VR therapy could stimulate patients’ attention and motivation, making the intervention more effective than conventional physical therapy (CPT). Therefore, the main objective of this systematic review and meta-analysis was to evaluate the effectiveness of VR interventions in the recovery of ULMF in patients with SCI.

## Methods

### Search Strategy

The PRISMA (Preferred Reporting Items for Systematic Reviews and Meta-Analyses) guidelines [30] were followed to perform this systematic review. The search protocol was registered in the International Prospective Register of Systematic Reviews (PROSPERO) database (CRD 42018093855). The literature search was performed between October and December 2019 in the following electronic databases: Embase, Web of Science, Cumulative Index to Nursing and Allied Health Literature (CINAHL), Scopus, Medline, Physiotherapy Evidence Database (PEDro), PubMed, and Cochrane Central Register of Controlled Trials (CENTRAL). The following descriptor terms combined with Boolean operators were employed: (“spinal cord injuries” OR “spinal cord injury” OR “quadriplegia” OR “paraplegia” OR “tetraplegia”) AND (“virtual reality exposure therapy” OR “virtual reality” OR “augmented reality” OR “virtual systems” OR “video games” OR “videogame” OR “exergaming” OR “exergames” OR “commercial games” OR “play-based therapy”). Medical Subject Headings (MeSH) descriptors were used in PubMed database: “virtual reality exposure therapy,” “virtual reality,” “spinal cord injuries,” and “video games.” The search was filtered to include full-text clinical trials papers. No date or language filters were applied.

### Selection Criteria

The Population, Intervention, Comparison, Outcomes and Study (PICOS) design model was used to establish the article inclusion criteria: (1) population: adults with SCI; (2) intervention: VR interventions; (3) comparison: adults with SCI performing CPT; (4) outcome: outcomes specifically related to ULMF, such as muscle strength, range of motion (ROM), dexterity, grasp and pinch force, and hand function; and (5) study design: controlled clinical trials. Articles of studies which included participants with SCI and other pathologies but did not provide the outcome data for each specific population were excluded.

### Study Selection Process and Data Extraction

The literature search was carried out by combining keywords in the scientific databases mentioned above and duplicated articles were excluded. Next, titles and abstracts were reviewed, and we excluded those articles that did not meet the established inclusion criteria. The remaining articles were analyzed strictly and were finally included in the systematic review. Two reviewers (ADM-R and MDR) took part independently in the study selection process, review, and systematic data extraction. A third reviewer (DLA) took part in achieving consensus in cases of dispute. The following data were extracted from the studies: (1) author and date of publication; (2) number and age of participants, levels of injury, and mean time post onset; (3) and characteristics of the interventions (intervention types in each group, outcome measures, measuring instrument) and results.

### Tools for Assessing the Risk of Bias of the Studies

The Cochrane Collaboration’s tool [31] and Review Manager (RevMan) software (version 5.3; The Nordic Cochrane Centre, The Cochrane Collaboration, 2014), which includes a description and evaluation of each item by means of a bias table, were used to assess the risk of bias. After assessing the risk of bias of each study, the studies were categorized as “low risk,” “high risk,” or “unclear risk.” Two reviewers carried out the assessment independently. In cases of doubt, the final decision was determined through discussion including a third reviewer.

### Statistical Analysis

The meta-analysis compared CPT with VR interventions. The studies were divided into subgroups based on the measuring instrument that was used in the study. If more than one instrument was used in the same study, we included the study in more than one subgroup. The differences in the effect size (postintervention minus preintervention) between the groups were analyzed in terms of the standardized mean difference. The confidence level was set at 95% (significance at *P*<0.05). Results are shown along with 95% CIs.

The chi-square test and the *I*^2^ statistic (percentage of variation across studies that is due to heterogeneity) were used to test the homogeneity, using a fixed-effect model in the case of homogeneity and a random-effects model otherwise.

The analyses were performed using RevMan 5.3 software, and the results are presented in tables and forest plots.

## Results

**Figure 1:**
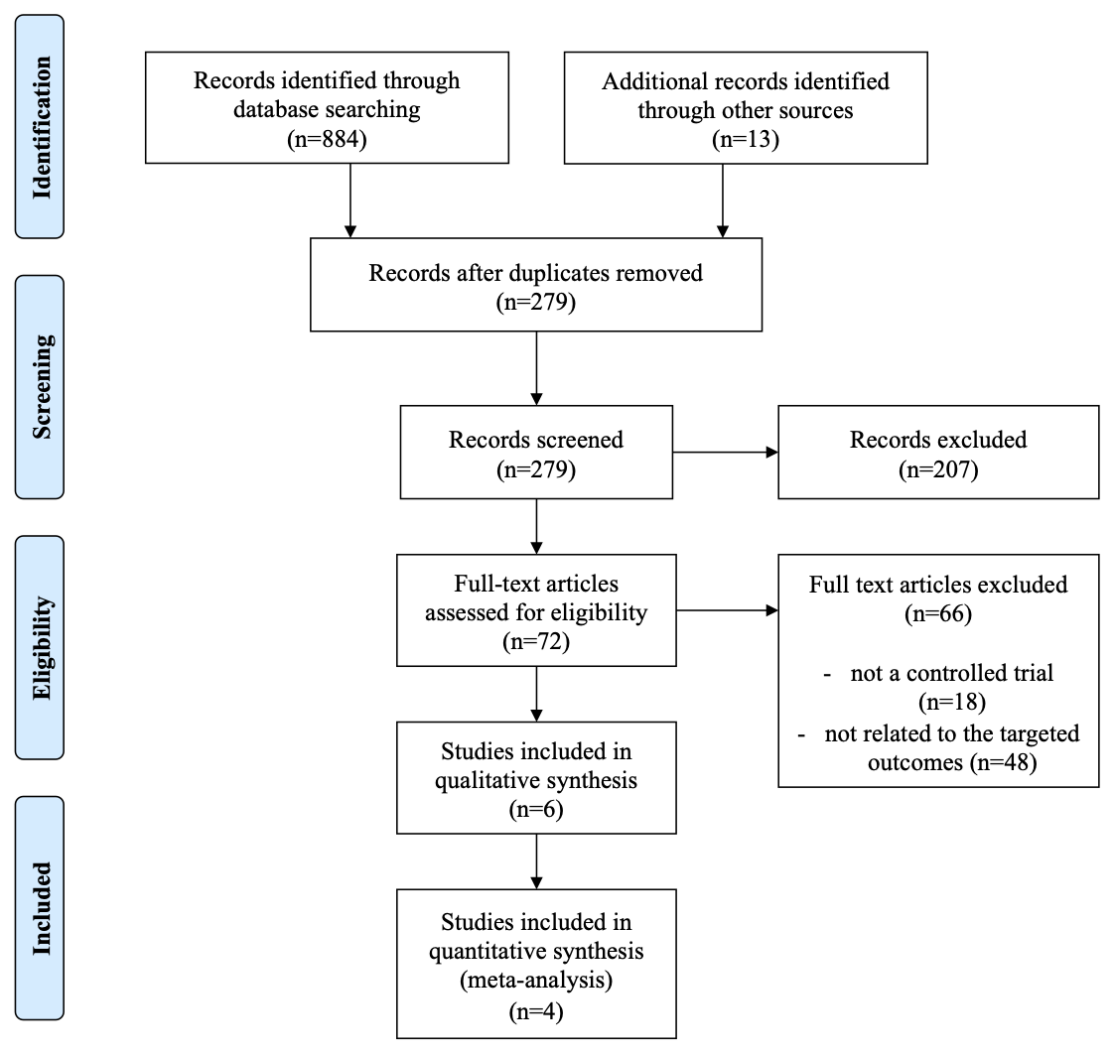
Information flow diagram of the selection process of the systematic review and meta-analysis.

A total of 279 potentially relevant articles were retrieved after the selection process, as shown in Figure 1. A total of 6 studies were included in the systematic review. Four of them were included in the meta-analysis for statistical comparison.

### Assessment of the Risk of Bias of the Studies Included in the Review

**Figure 2:**
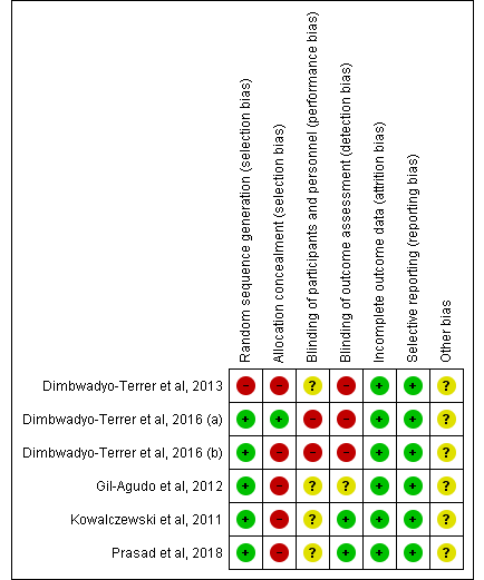
Risk of bias of the studies included in the systematic review. The green circle (+) indicates low risk of bias, the yellow circle (?) unclear risk of bias, and the red circle (-) high risk of bias.

**Figure 3:**
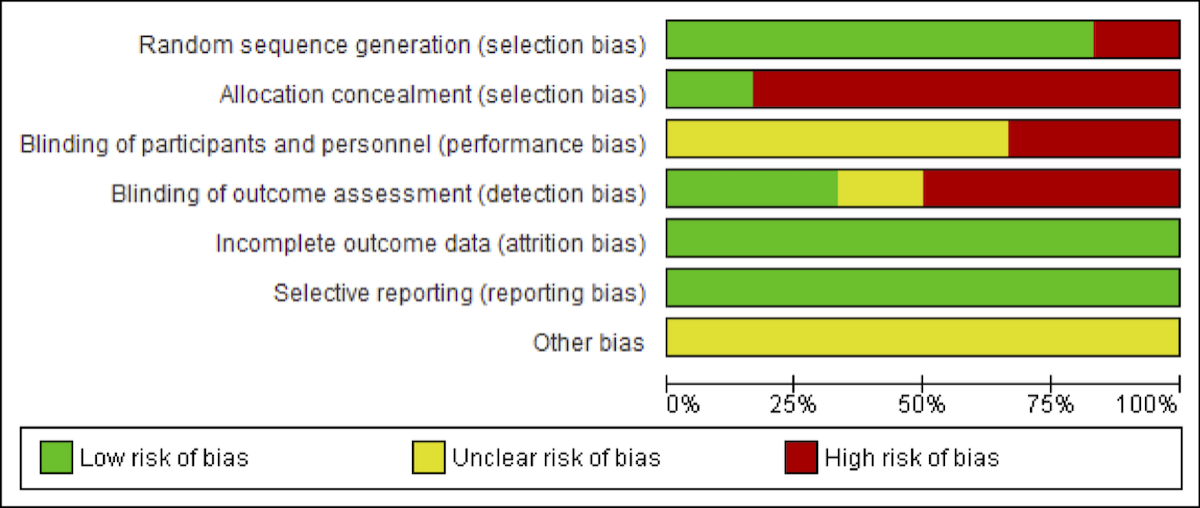
Overall risk of bias, with each category presented as percentages.

Regarding the risk of bias of the studies included in this review, the studies conducted by Kowalczewski et al [32], Dimbwadyo-Terrer et al [3], and Prasad et al [33] presented the lowest risk of bias, as shown in Figure 2. Furthermore, concerning the risk of bias among the studies analyzed, the lowest biases were found in the selective reporting (0%) and the incomplete outcome data (0%). The highest value (85.5%) was found in the allocation concealment, as shown in Figure 3.

### Data Extraction

A total of 103 subjects (control group, n=46; intervention group, n=57) took part in the different studies. A study by Dimbwadyo-Terrer et al [3] had the highest number of participants (n=31) and a different study by Dimbwadyo-Terrer et al [34] had the lowest sample size (n=9). The mean age of the participants ranged from 23.7 years [33] to 54.3 years [34]. Concerning the neurological level of injury, 4 studies [3,8,32,35] included participants with American Spinal Injury Association Impairment Scale (AIS) grades A-B injuries, while 2 studies [33,34] included participants with AIS grades A-D injuries. The main characteristics of the participants are shown in Table 1.

**Table 1:** Main characteristics of the participants in each study.

	Participants, n						
Study reference	Total	CG^a^	IG^b^	Age (years), mean (SD)	AIS^c^ grade	Level of injury	Mean time after disease onset (months)
[32]	13	7	6	Overall: 35.9 (11.9)	A-B	C5-C7	Overall: 9.0
[8]	10	5	5	CG: 49.0 (6.1)	A-B	C5-C8	CG: 5.8
				IG: 36.2 (10.4)			IG: 4.2
[35]	18	6	12	CG: 42.0 (13.6)	A-B	C5-C8	CG: 3.6
				IG: 33.6 (14.1)			IG: 6.6
[3]	31	15	16	CG: 40.2 (13.6)	A-B	C5-C8	CG: 5.6
				IG: 34.5 (13.7)			IG: 4.3
[34]	9	3	6	CG: 44.2 (22.9)	A-D	T1-T6	CG: 5.0
				IG: 54.3 (9.9)			IG: 5.8
[33]	22	10	12	CG: 33.9 (7.1)	A-D	C5-C8	CG: 10.2
				IG: 23.7 (5.2)			IG: 15.2

^a^CG: control group.

^b^IG: intervention group.

^c^AIS: American Spinal Injury Association Impairment Scale.

Concerning the intervention protocols, the VR therapy was applied to the intervention groups via different technological devices while the comparison group performed CPT. The longest total duration of intervention and the highest intensity were achieved by Kowalczewski et al [32] (5 times/week for 6 weeks). In addition, the longest session duration (60 minutes) was achieved by Kowalczewski et al [32] and Prasad et al [33]. Conversely, the shortest intervention time and lowest intensity were achieved by Dimbwadyo-Terrer et al [34], who only performed 4 sessions (2 times/week for 2 weeks).

VR therapy was provided through different devices, such as the Rehabilitation Joystick for Computerized Exercise (ReJoyce) VR system (Saebo Inc) [32], Toyra system (National Paraplegics Hospital of Toledo and Rafael del Pino Foundation) [3,8,35], Nintendo Wii [33], and a mesh data glove [34]. All of the devices used to provide VR therapy were categorized into semi-immersive and nonimmersive VR types. The ReJoyce VR system consists of a workstation where patients can play games shown on a screen through a segmented, jointed, spring-loaded arm. The Toyra system was used in 3 studies [3,8,35]. It reproduces the patient movements in real time through an avatar displayed on the screen, and patients can interact with different objects in the virtual environment [8]. Prasad et al [33] used the Nintendo Wii video game console. Finally, the study by Dimbwadyo-Terrer et al [34] used a data glove to interact with the virtual environment, allowing patients to manipulate virtual objects in real time.

Regarding the different deficits treated, all of the studies analyzed the effects of the VR intervention on ULMF. Moreover, the authors focused their interventions on improving upper limb ROM [8,32,35], upper limb strength [8], upper limb dexterity [33], grasp and pinch force [32], and functional performance [3,8,33-35]. Most studies reported no significant effects on the different outcomes analyzed. Only the study of Kowalczewski et al [32] showed benefits on all of the outcomes. Gil-Agudo et al [8] showed significant results on stacked checked subtest of the Jebsen-Taylor Hand Function Test (JTHFT) [36], and Dimbwadyo-Terrer et al [3] got significant benefits for muscle strength measured by the muscle balance test [37]. Table 2 shows the main characteristics of the different interventions performed by the different studies.

**Table 2:** Main characteristics of the interventions.

Study	Group interventions	Intensity	Session duration	Intervention duration	Outcomes	Measuring instrument	Results
[32]	CG^a^: CPT^b^; IG^c^: ﻿ReJoyce VR^d^ system	5 x/wk^e^	60 min^f^	6 wks^g^	Upper limb motor function, ROM^h^, functional tasks, grasp, and pinch forces	ARAT^i^, ReJoyce automated hand function test	All outcomes showed statistically significant differences and clinically important improvements for IG.
[8]	CG: CPT; IG: Toyra VR system	3 x/wk	30 min	5 wks	Upper limb ROM, motor function and strength, and functional performance	NHPT^j^, JTHFT^k^, MI^l^, BI^m^, FIM^n^, SCIM^o^	No significant differences were found between groups after intervention, except for JTHFT stacking checkers subtest (*P*=0.008).
[35]	CG: CPT; IG: Toyra VR system	4 x/wk	ND^p^	3 wks	Upper limb ROM and motor function, and functional performance	MI, MB^q^, FIM, SCIM	No significant differences were found between groups after intervention.
[3]	CG: CPT; IG: Toyra VR system	3 x/wk	30 min	5 wks	Upper limb motor function and functional performance	MB, MI, FIM, SCIM, BI	No significant differences were found between groups after intervention. At follow-up, only MB was statistically improved (*P*=0.04).
[34]	CG: CPT; IG: VR system + CiberTouch data glove	2 x/wk	30 min	2 wks	Upper limb motor function and functional performance	NHPT, JTHFT, MB, SCIM	No significant differences were found between groups after intervention.
[33]	CG: CPT; IG: Nintendo Wii	3 x/wk	60 min	4 wks	Upper limb dexterity and motor function, and functional performance	CUE^r^, BBT^s^, SCIM, WHOQOL-BREF^t^	No significant differences were found between groups after intervention.

^a^CG: control group.

^b^CPT: conventional physical therapy.

^c^IG: intervention group.

^d^VR: virtual reality.

^e^x/wk: times/week.

^f^min: minutes.

^g^wks: weeks.

^h^ROM: range of motion.

^i^ARAT: Action Research Arm Test.

^j^NHPT: Nine-Hole Peg Test.

^k^JTHFT: Jebsen-Taylor Hand Function Test.

^l^MI: Motricity Index.

^m^BI: Barthel Index.

^n^FIM: Functional Independence Measure.

^o^SCIM: Spinal Cord Independence Measure.

^p^ND: not described.

^q^MB: muscle balance.

^r^CUE: Capabilities of Upper Extremity.

^s^BBT: Box and Block Test.

^t^WHOQOL-BREF: World Health Organization Quality of Life Scale, Abbreviated Version.

### Instruments of Measurement Used in the Meta-Analysis

Different scales and tests were used in the studies to assess ULMF. The Nine-Hole Peg Test (NHPT) involves placing and removing pegs into 9 holes, and scores are based on the time taken to complete the activity. This scale is commonly used to measure fine manual dexterity [38]. Muscle balance (MB) tests are used to rate muscle strength, assigning a grade from 0 to 5 according to the strength of the muscle to face the gravity or the force applied by the examiner [37]. The Motricity Index ﻿(MI) measures the range and strength of active movements and each movement is rated on a point scale from 0 to 100 [39]. The JTHFT assesses the time (in seconds) spent to perform different tasks related to hand functioning, which are commonly used in activities of daily living, and it comprises 7 subtests (writing, simulated page turning, picking up small common objects, simulated feeding, stacking checkers, picking up large light objects, and picking up large heavy objects) [36]. Finally, ROM tests consist of measuring joint mobility using a goniometer [40]. A total of 4 studies were included in the meta-analysis.

**Figure 4:**
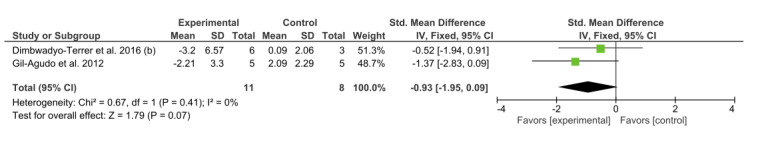
Forest plot for upper limb motor function measured by the Nine-Hole Peg Test. The green blocks indicate the weight assigned to the study, the horizontal line depicts the CI, and the black rhombus shows the overall result. IV: inverse variance; Std: standard.

Two studies [8,34] used the NHPT to analyze ULMF. According to the *I*^2^ statistic, 0% of variation across studies was due to heterogeneity. This homogeneity was confirmed by the chi-square test (*P*=0.41). A fixed-effect model was fitted. The study by Gil-Agudo et al [8] obtained the best results. We observed that VR therapy turned out to be more effective than CPT. However, the overall result of this meta-analysis was not conclusive, as shown in Figure 4.

**Figure 5:**
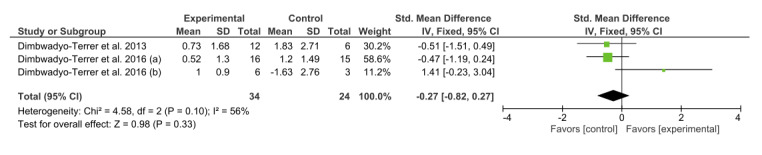
Forest plot for upper limb motor function measured by the muscle balance test. The green blocks indicate the weight assigned to the study, the horizontal line depicts the CI, and the black rhombus shows the overall result. IV: inverse variance; Std: standard.

Three studies [3,34,35] analyzed the effects of VR interventions using the results obtained from the MB test. In this group, *I*^2^=56%, although the chi-square test (*P*=0.10) showed homogeneity, and a fixed-effect model was fitted. Favorable results for VR interventions were obtained in the study by Dimbwadyo-Terrer et al [34]. However, none of these results were statistically significant. The overall result of this meta-analysis was not conclusive, as shown in Figure 5.

**Figure 6:**
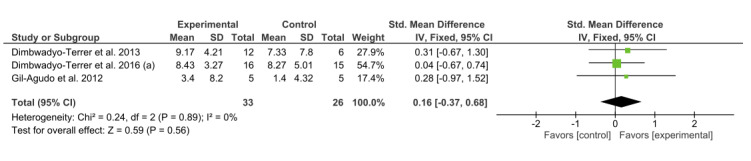
Forest plot for upper limb motor function measured by the Motricity Index. The green blocks indicate the weight assigned to the study, the horizontal line depicts the CI, and the black rhombus shows the overall result. IV: inverse variance; Std: standard.

Three studies [3,8,35] used the MI to assess ULMF. As with the studies that used the NHPT to analyze ULMF, 0% of the variation across studies was due to heterogeneity (*I*^2^=0%), and the chi-square test confirmed that finding (*P*=0.89). A fixed-effect model was fitted. All the studies showed favorable results for VR interventions. However, none of these results were statistically significant. The overall result of this meta-analysis was not conclusive, as shown in Figure 6.

**Figure 7:**
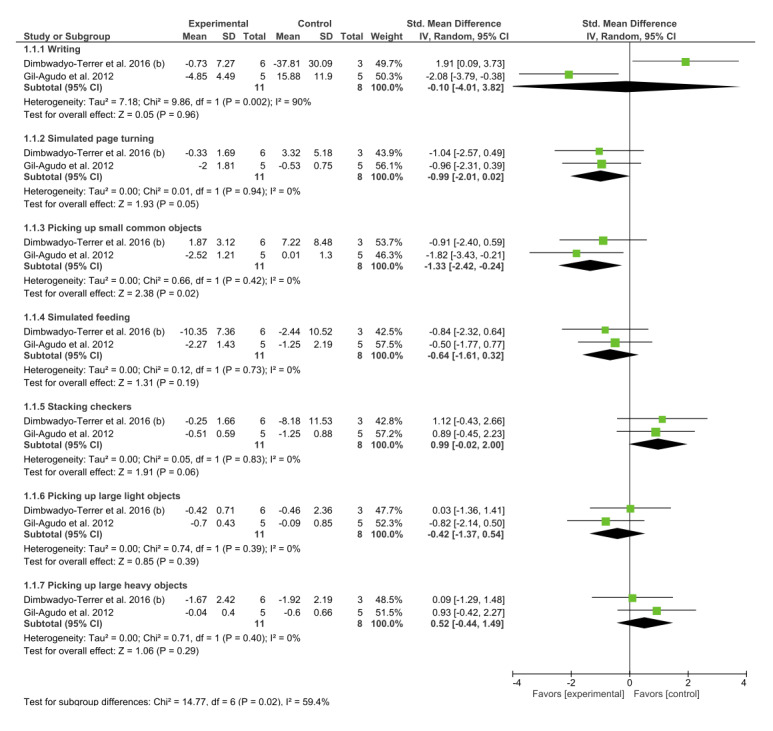
Forest plot for upper limb motor function measured by the Jebsen-Taylor Hand Function Test. The green blocks indicate the weight assigned to the study, the horizontal line depicts the CI, and the black rhombus shows the overall result. IV: inverse variance; Std: standard.

The JHFT was used to measure the ULMF in two studies [8,34]. The results suggested statistically significant results for VR interventions in the “picking up small common objects” subgroup. The overall result for the remaining subgroups was not conclusive (Figure 7).

**Figure 8:**
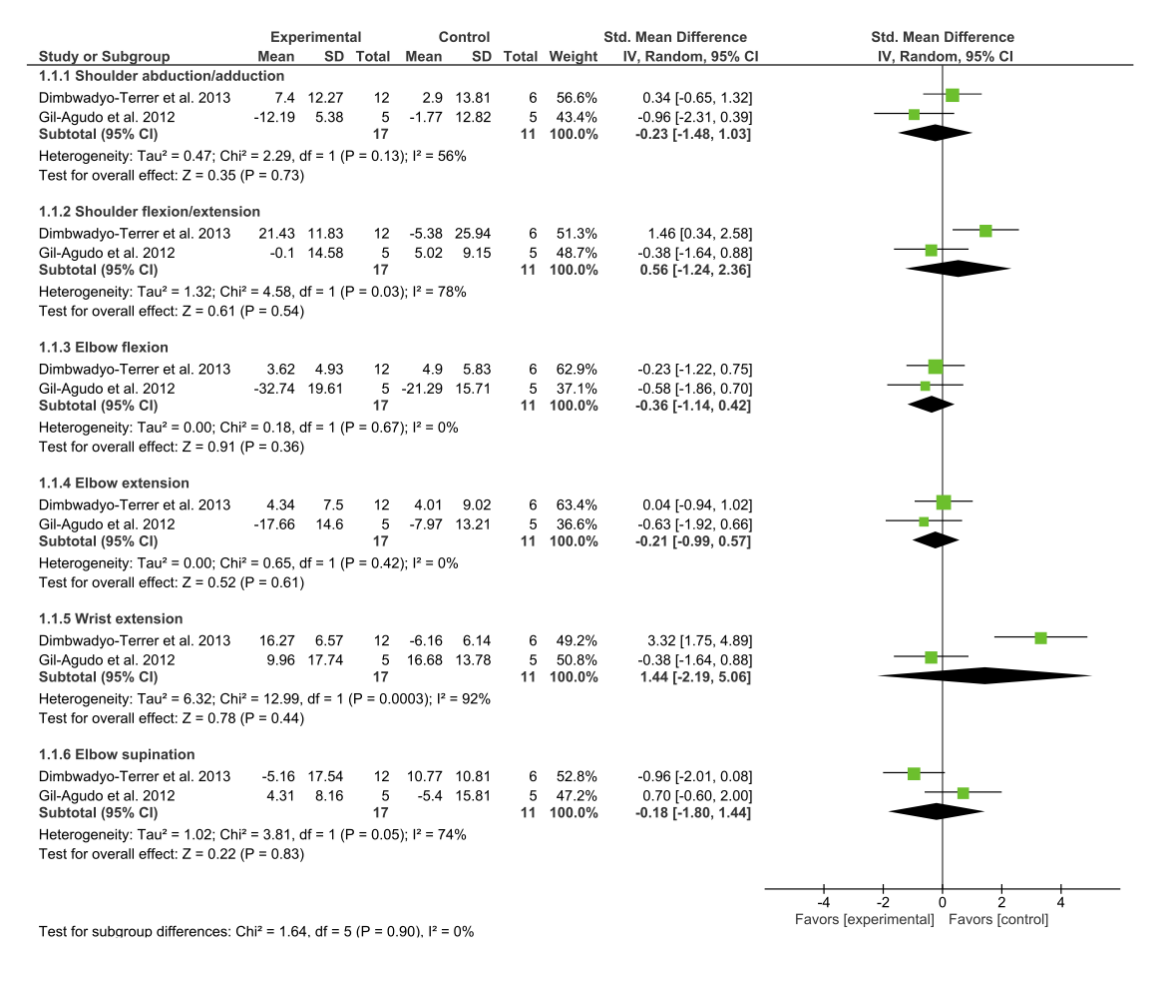
Forest plot for upper limb range of motion. The green blocks indicate the weight assigned to the study, the horizontal line depicts the CI, and the black rhombus shows the overall result. IV: inverse variance; Std: standard.

Finally, the ROM was measured in 2 of the studies [8,35]. None of the subgroups in this meta-analysis led to conclusive results, as shown in Figure 8.

## Discussion

### Principal Findings

The present research aimed to use meta-analysis to evaluate the effectiveness of VR versus CPT on ULMF in patients with SCI. Six controlled trials were included in the systematic review and 4 of them were included in the meta-analysis. A total of 105 participants were involved in the different studies. In view of our results, we can conclude that there is not enough evidence that VR interventions are more effective than CPT in helping patients to recover ULMF after SCI.

These results match with those obtained in our previous meta-analysis [29] on functional performance recovery in patients with SCI. Furthermore, from the 6 studies included in this review, 5 [3,8,33-35] analyzed the effects of VR therapy on functional performance and none of them showed significant results. According to the International Classification of Functioning, Disability and Health (ICF), we can hypothesize that ULMF impairments influence the loss of functional performance, since impairments at the body structure and functional level can influence activity limitations and participation restrictions [41]. Conversely, our results do not match with those of Yeo et al [28] and de Araújo et al [27], who reported positive effects of VR interventions on motor function, but the reviews were not restricted specifically to assess ULMF. We suggest that the inconclusive results on ULMF revealed in the present review could have been affected by the type of VR devices used in the interventions. All of the studies performed the VR interventions through semi-immersive or nonimmersive systems, where a computer or video game console displayed the virtual environment through a screen [41]. None of the studies used immersive VR devices, which could provoke more task-focused attention than semi-immersive and nonimmersive devices [42]. Additionally, other heterogeneous factors could have influenced the results obtained, such as different tasks being performed in the VR sessions, different protocols being used for VR interventions and CPT, different session and program durations, and the participants’ characteristics. Therefore, it would be desirable to unify protocols in order to clarify which factors of VR interventions may be more appropriate to achieve the intended effects.

Concerning the characteristics of participants, the injury severity was measured by AIS grades. Most studies [3,8,32,35] included participants with AIS grades A-B, while 2 studies [33,34] included AIS grades A-D. Regarding the levels of injury, most studies [3,8,32,33,35] included patients with cervical levels of injury. Although several positive effects were found in patients with AIS A-B grades and cervical levels, we cannot conclude that the recovery of ULMF is related to the level of injury. Regarding the different effects obtained in the studies, of the 6 studies included in the present review, only the study by Kowalczewski et al [32] showed significant improvements in ULMF, ROM, functional tasks, grasp, and pinch forces. These improvements might have been seen in the study because the intervention had the longest total duration and a higher intensity (60 minutes, 5 times/week for 6 weeks).

Although VR systems have the potential to provide precise measurement of motor outcomes, provide direct feedback and safe environments [13,43], and increase patient motivation and treatment adherence [8] in clinical settings, we did not find differences between VR interventions and CPT in improving ULMF in patients with SCI. According to Morone et al [44], further research is needed in order to develop accurate user guidelines before VR systems are ready for market, to develop immersive VR systems based on personalized neurological characteristics optimizing motor learning processes [45], to implement adequate training to health care professionals [46], and to integrate this technology into neurological rehabilitation [47].

### Limitations and Recommendations for Future Research

Some limitations of the study should be mentioned. The results provided by the present review should be viewed with caution because of the limited number of controlled trials analyzed. Another limitation was the small sample size used in the studies and the different injury levels of the patients. Thus, we encourage authors to use large sample sizes and to include an appropriate number of subjects in stratified groups in order to know which factors of the participants’ characteristics are influencing the results. Nevertheless, these patients are usually treated in neurological institutions or centers and it is difficult to get large sample sizes. Thus, most studies include convenience samples, resulting in potential selection biases [48]. Furthermore, the heterogeneous protocols used in terms of VR devices employed, program and session durations, and CPT protocols used could affect the results obtained in this review.

In this sense, we encourage researchers to perform randomized controlled trials with higher methodological quality using larger sample sizes, and to unify VR intervention protocols in order to identify the key aspects of VR interventions that have the greatest impact on ULMF recovery after SCI. In addition, because task-focused attention is stimulated more with immersive VR devices than with semi-immersive and nonimmersive devices [42] and no positive results on ULMF were obtained using semi-immersive and nonimmersive VR devices in the studies analyzed in this review, we encourage researchers to use immersive VR devices in their future clinical trials. Finally, we urge researchers to analyze the effectiveness of the application of different CPT techniques in patients with SCI in order to provide further evidence for this topic.

### Conclusions

Although the use of VR devices has expanded in neurological rehabilitation, the current evidence for using VR interventions to improve ULMF in patients with SCI is limited. Specifically, our results showed that VR may not be more effective than CPT in ULMF recovery. This may be explained by the fact that all the studies used semi-immersive and nonimmersive devices, and these devices require less task-focused attention than immersive VR devices. No solid conclusions can be drawn concerning the relationship between injury levels and severity and the effects of VR interventions.

In view of our results, it is necessary to conduct clinical trials with a high methodological quality, using larger sample sizes, and to unify VR intervention protocols in order to identify the key aspects that increase the clinical impact of VR interventions in neurological rehabilitation. Further research is needed to provide evidence for the application of VR devices to facilitate ULMF recovery in patients with SCI.

## Abbreviations

AIS: American Spinal Injury Association Impairment Scale
CENTRAL: Cochrane Central Register of Controlled Trials
CINAHL: Cumulative Index to Nursing and Allied Health Literature
CPT: conventional physical therapy
ICF: International Classification of Functioning, Disability and Health
JTHFT: Jebsen-Taylor Hand Function Test
MeSH: Medical Subject Headings
MI: Motricity Index
NHPT: Nine-Hole Peg Test
PEDro: Physiotherapy Evidence Database
PICOS: Population, Intervention, Comparison, Outcomes and Study
PRISMA: Preferred Reporting Items for Systematic Reviews and Meta-Analyses
PROSPERO: International Prospective Register of Systematic Reviews
ROM: range of motion
SCI: spinal cord injury
ULMF: upper limb motor function
VR: virtual reality
